# Influence of Altitudes and Development Stages on the Chemical Composition, Antioxidant, and Antimicrobial Capacity of the Wild Andean Blueberry (*Vaccinium floribundum* Kunth)

**DOI:** 10.3390/molecules27217525

**Published:** 2022-11-03

**Authors:** Mabel Guevara-Terán, Katherine Padilla-Arias, Andrea Beltrán-Novoa, Ana M. González-Paramás, Francesca Giampieri, Maurizio Battino, Wilson Vásquez-Castillo, Paulina Fernandez-Soto, Eduardo Tejera, José M. Alvarez-Suarez

**Affiliations:** 1 Grupo de Bioquimioinformática, Universidad de Las Américas, Quito 170125, Ecuador; 2 Grupo de Investigación en Polifenoles, Campus Miguel de Unamuno, Universidad de Salamanca, 37008 Salamanca, Spain; 3 Facultad de Postgrado, Universidad de Las Américas, Quito 170125, Ecuador; 4 Departamento de Química Analítica, Facultad de Ciencia y Tecnología, Universidad del País Vasco/Euskal Herriko, Unibertsitatea (UPV/EHU), 48940 Bilbao, Spain; 5 Research Group on Food, Nutritional Biochemistry and Health, Universidad Europea del Atlántico, 39011 Santander, Spain; 6 Department of Biochemistry, Faculty of Sciences, King Abdulaziz University, Jeddah 2254, Saudi Arabia; 7 Department of Clinical Sciences, Università Politecnica delle Marche, 60121 Ancona, Italy; 8 International Research Center for Food Nutrition and Safety, Jiangsu University, Zhenjiang 212013, China; 9 Ingeniería Agroindustrial, Universidad de Las Américas, Quito 170125, Ecuador; 10 Departamento de Ingeniería en Alimentos, Colegio de Ciencias e Ingenierías, Universidad San Francisco de Quito, Quito 170901, Ecuador

**Keywords:** Andean blueberry, altitude, ripeness, chemical composition, antioxidant capacity, antimicrobial activity

## Abstract

The chemical composition and biological capacities of berries depend on environmental parameters, maturity, and location. The Andean blueberry (*Vaccinium floribundum* Kunth), also known as mortiño, presents a unique combination of several phytochemicals, which play a synergistic role in its characterization as a functional food. We aimed to expose the possible variations that exist in the profile of the phenolic compounds as well as the antioxidant and antimicrobial capacity of the wild Andean blueberry with respect to three ripeness stages and two different altitudes. We found that polyphenols are the predominant compounds in the berry during the early ripeness stage and are the main bioactive compounds that give rise to the antioxidant capacity and inhibition effect on the growth of gram-positive and gram-negative bacteria. Moreover, the accumulation of ascorbic acid, free amino acids, and anthocyanins increases as the ripening process progresses, and they were the main bioactive compounds in the ripe berry. The latter compounds influence the production of the typical bluish or reddish coloration of ripe blueberries. In addition, it was determined that environmental conditions at high altitudes could have a positive influence in all cases. Overall, our data provide evidence regarding the high functional value of the wild Andean blueberry.

## 1. Introduction

Forest fruits or red fruits, commonly known as berries, are a group of mostly edible fruits that are distributed in several taxonomic families, the Ericaceae family being one of the most studied [1,2]. The genus *Vaccinium* belongs to this family and includes more than 400 species, including *Vaccinium floribundum* Kunth, also known as the Andean blueberry or “mortiño”, an endemic species of the Andean region of northwestern South America [3,4].

The Andean blueberry contains a unique combination of several phytochemicals, nutrients, and fiber, which play a synergistic role in its characterization as a functional food [3,5]. However, few studies have described its high biological potential in humans [5]. Blueberries (Vaccinium spp.) have been identified as one of the five healthy foods certified by the International Food and Agriculture Organization (FAO) due to their high phenolic compound and nutrient content [6]. These fruits have been characterized by high concentrations of flavonoids with potential use in the pharmaceutical, nutraceutical, and food industries [7,8]. There are few reports documenting their phenolic profile. However, from the available studies it has been possible to determine ellagitannins, ellagic acids, anthocyanins, flavonol glycosides, flavan-3-ols, and hydroxycinnamics as the main compounds [3,8,9,10] that act as antioxidants in the human body and perform anticarcinogenic, anti-inflammatory, antihypertensive, antimutagenic [1,11,12], and antibacterial functions [13,14].

Epidemiological studies suggest that the regular consumption of blueberries reduces the risk of cardiovascular disease, type 2 diabetes mellitus, and neurological deterioration [15]. Blueberries have been shown to improve cognitive and memory functions in the elderly [16], and polyphenol-rich extracts from these berries have neuroprotective potential, increasing the viability rate of human neural progenitor cells and protecting them from stressors [17]. In addition, the phenolic compounds in blueberries protect against metabolic disorders, reduce hyperglycemia, and act on the gastrointestinal microflora, contributing to the health of the host, which has implications for degenerative diseases and aging [6,17].

In Ecuador, *V. floribundum* Kunth is a wild species grown in isolation or in groups with other species in high inter-Andean valleys and paramos between 2200 and 3400 m above sea level [2,3,4]. The fruit measures approximately 5–10 mm in diameter [5] and its color varies from green to dark purple during ripening [11]. The harvest period of the fruits must be carried out when they are 100% ripe (red), which means they have good firmness, a higher concentration of total soluble solids, low acidity (2.7%), and a pH of 2.1 [18]. The species’ commercial use is still low [7]. However, it is sold at local markets during two ripening seasons: one between April and May and the other between September and December. It is used to make jellies, jams, sauces, and even wine and boiled drinks, such as “colada morada” [1,3,4,10].

Plant growth, fruit quality and yield are closely associated with environmental conditions, which can be adversely affected by unfavorable weather conditions [19]. Furthermore, scientific studies have shown that the chemical composition and biological capacities of berries of the genus *Vaccinium* depend on environmental parameters, maturity, location [1,14] and storage technology [20,21].

In fact, anthocyanins have been identified as the main flavonoid involved in fruit ripening [9]. Currently, it is known that these compounds influence the production of the bluish or reddish coloration typical of the ripe fruits. The metabolic synthesis of flavonoids is well described for some species; however, the synthesis and concentration of anthocyanins in *V. floribundum* Kunth in terms of the ripening process are still unknown [14]. On the other hand, it has been shown that climatic trends, such as total atmospheric pressure, increasing radiation under cloudless skies, and reduction of atmospheric temperature, are associated with altitude, and the combined action of these variables could play a role in determining the final phenolic profile of fruits [22,23,24].

Considering the importance of knowing how external factors such as altitude gradients and ripeness stages can affect the characteristics of the wild Andean blueberry, the aim of this article was to expose the possible variations that exist in the profile of the phenolic compounds as well as the antioxidant and antimicrobial capacity of the wild Andean blueberry with respect to both variables.

## 2. Materials and Methods

### 2.1. Collection of Wild Andean blueberries

The wild Andean blueberries (*V. floribundum* Kunth) were collected during their corresponding crop seasons between April and November 2021. Two provinces were selected for the collection of the fruits: Imbabura, located at 3641 m above sea level (m.a.s.l.), and Carchi at 2836 m.a.s.l. We considered (i) the abundance of the populations of this plant, (ii) the level of fruit production, and (iii) the altitude difference between the populations. Both provinces are in the inter-Andean corridor of Ecuador, and the plants grow in the well-known Ecuadorian highlands. The specimens were identified by Prof. Wilson Vázquez (Universidad de Las Américas (UDLA), Ecuador) using the reference specimens deposited in the UDLA research laboratories.

**Figure 1:**
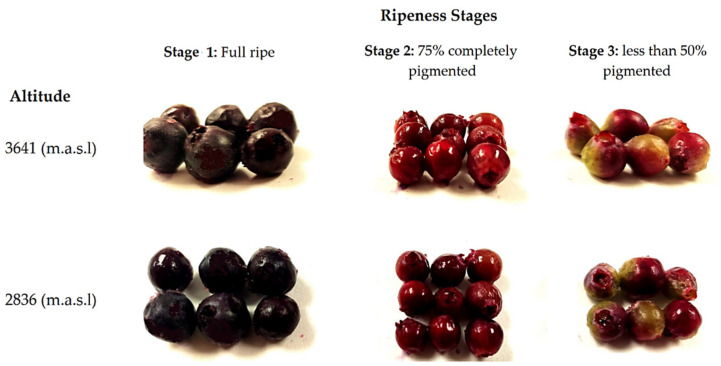
Wild Andean blueberries (*V. floribundum* Kunth) collected at three stages of ripeness based on their pigmentation at two different altitudes.

In each zone, three batches of 0.1 kg of fruits without damage (physical, physiological, or pathogenic) were randomly collected from different plants. We also considered the stage of ripeness based on their pigmentation (stage 1: fully ripe, stage 2: 75% to completely pigmented, and stage 3: less than 50% pigmented). During fruit ripening, color changes occurred: black when it is ripe for consumption (stage 1), pink when it is physiologically mature (stage 2), and green when it is not yet ripe (stage 3) [25]. No differences were identified in terms of the size and shape of the fruits from the same stage of maturity between both altitudes, as shown in Figure 1. The fruits were carefully transported from the harvest site to the laboratory in a period of no longer than 24 h (3 h for fruit harvesting in Zuleta and no more than 5 h for fruit harvesting in La Paz). Once in the laboratory, the fruits were selected by removing those that suffered any damage during transport (e.g., drained or broken). Next, the selected fruits from different plants collected at the same altitude and at the same stage of ripeness were pooled (6 pools were formed: three stages and two altitudes) and immediately frozen for 48 h at −80 °C. After this process, the pools were lyophilized and ground to a fine powder using an analytical mill (IKA A11 basic, Germany). The pools’ powder was kept in a vacuum in the dark and at a temperature of −80 °C until compositional analysis (no more than 4 months from its preparation). Before the analysis, three independent extractions were made from each pool.

Each collection area was georeferenced (Table 1), and the environmental conditions were considered (Table 2 and Supplementary Material). The values in Table 2 correspond to the average, minimum and maximum values recorded every 2 h over a period of 10 days prior to harvest, while in Supplementary Material it is presented in a graphical form with the entire records.

### 2.2. Preparation of Hydroalcoholic Extracts

Hydroalcoholic extracts were obtained according to previously reported methods [26,27]. Powder samples (1 g) were extracted with methanol-water (80:20) (40 mL), and continuously shaken for 2 h, and protected from light at room temperature. Next, samples were centrifuged for 10 min at 5000 rpm (10 °C) and the supernatant filtered through a 0.45 μm Minisart filter (RephiLe Bioscience Ltd., Acton, MA, USA). The solid residue was re-extracted twice with the same volume, and supernatants were combined. Finally, the extracts were dried in a vacuum at 40 °C and the dry residues were stored at −20 °C until further analysis.

### 2.3. Spectrophotometric Determination of Reducing Capacity, Flavonoid Anthocyanin, and Free Amino acid Contents

Reducing capacity, anthocyanins, flavonoid, and free amino acid contents were determined using the hydroalcoholic extracts. The Folin-Ciocalteu method [28] was used to determine the RC and the results were expressed as milligrams of gallic acid equivalents (GAE) (0.1875–1.0 mM, y = 0.9418x + 0.053, R^2^ = 0.9821) per 100 g of fresh weight (FW) of fruit (mg GAE/100 g FW). The pH differential method [29] was used to determine the total anthocyanin (ACY) and the results were expressed as milligrams of Pg-3-gluc equivalents (PgEq) (40–150 mg/mL, y = 0.0067x − 0.0627, R^2^ = 0.9949) per gram of FW of fruit (mg PgEq/g FW). Total flavonoid content (TFC) was determined using the aluminum chloride method [30] and the results were expressed as milligrams of catechin equivalents (Cateq) (0.02–0.308 mg/mL, y = 1.8779x − 0.002, R^2^ = 0.9987) per gram of FW of fruit (mg Cateq/g FW), while free amino acids (FAAs) were quantified using the Cd-ninhydrin method [31], and the results were expressed as mg of Leucine equivalents (LEeq) (2.4–42 mg/mL, y = 0.017x + 0.1289, R^2^ = 0.976) per 100 g of FW of fruit (mg LEeq/g FW).

### 2.4. HPLC-DAD Analysis of Ascorbic acid

Ascorbic acid was determined as previously reported [32]. Briefly, 5 mg of freeze-dried fruit powder was dissolved in 5 mL of the extraction solution (metaphosphoric acid 5% *w*/*v*), sonicated for 15 min, and filtered through a syringe filter (0.22 µm, RephiLe Bioscience Ltd., Acton, MA, USA). Immediately afterward, 20 μL were analyzed on an HPLC system (Agilent Technologies 1260 Series, Santa Clara, CA, USA) composed of a Diode Array Detector (DAD) (Agilent Technologies 1260 Infinity G1315C) set at an absorbance of 245 nm and a Quaternary Pump (Agilent Technologies 1260 Infinity G1312B). An Eclipse Plus C18 column (5 μm, 4.6 × 150 mm) was used as a stationary phase, while monopotassium phosphate (KH_2_PO_4_) (50 mM) was used for elution at a flow rate of 1 mL/min for 20 min. A calibration curve of ascorbic acid (AsA) (5.07–50.75 mg/L, y = 235.54x − 53.136, R^2^ = 0.9979) was used and the results were expressed as mg of AsA per g of FW (mg AsA/g FW).

### 2.5. Polyphenolic Profile Determination via HPLC-DAD/ESI-MS^n^

The hydroalcoholic extracts were dried in a vacuum until all the solvent was removed. The resulting crude extracts were dissolved in 1 mL of methanol-water solution (80:20, *v*/*v*) and injected into a UHPLC separation and dual online detection system using a diode array detector and mass spectrometry (MS).

The UHPLC system consisted of a Vanquish (Thermo Fisher Scientific, Waltham, MA, USA) fitted with a binary pump and DAD coupled with an LTQ-XL (Thermo Fisher Scientific, Waltham, MA, USA). Xcalibur Software was controlled. An Accucore Vanquish C18 column (1.5 μm, 100 × 2.1 mm) (Merck KGaA, Darmstadt, Germany) thermostated at 40 °C was used as a stationary phase, while for elution we used a solution of 0.1% formic acid: water (A) and 0.1% formic acid: acetonitrile (B). The elution gradient established was of the isocratic type 2% B for 4 min, 4% B from 4 min to 22 min, 40% B for 10 min, 70% B for 3 min, and 2% B for 5 min, and finally at 45 min the column was rebalanced to the initial conditions of the solvent. The flow rate was 0.2 mL/min. Double line detection was carried out in DAD at 220, 330, and 370 nm as preferred wavelengths, and MS operated in negative ion mode. The dependent data analysis (DDA) was performed on the 5 most intense ions with a normalized collision energy of 35.

Spectra between *m*/*z* 50 and *m*/*z* 1500 were recorded. The ESI conditions were a capillary temperature of 275 °C, source voltage and capillary voltage of 5 KV and −35 V, respectively, and tubeless −200. The phenolic compounds were tentatively identified from their UV-Vis and mass spectra.

### 2.6. Spectrophotometric Determination of Total Antioxidant Capacity

The total antioxidant capacity of the hydroalcoholic extracts was determined through the ferric reducing/antioxidant power (FRAP) (50–500 μM, y = 0.0.0017x + 0.003, R^2^ = 0.9988) assay [33] and the 2.2-diphenyl-1(2,4,6-trinitrophenyl) hydrazyl (DPPH radical) method [34] (50–950 μM, y = 0.1034x + 9.6775, R^2^ = 0.9704). The results of both methods were expressed as μmol of Trolox equivalents (TEq) per g of FW of plant (μmol TEq per g FW).

### 2.7. Bacterial Strains, Media, and Growth Conditions

The bacterial strains Enterococcus faecium ATCC27270, Enterococcus faecalis ATCC29212, Staphylococcus aureus ATCC25923, Klebsiella pneumoniae ATCC700603, Acinetobacter baumannii ATCC19606, Pseudomonas aeruginosa ATCC27853, Enterobacter cloacae ATCC23355, and Escherichia coli ATCC25922 were bought from ATTC and used in the antimicrobial activity tests of the extracts. Culture bacteria were grown in Nutrient Agar (NA) and overnight cultures were prepared in Nutrient Broth (NB). Unless stated otherwise, all cultures were incubated at 37 °C.

### 2.8. Antimicrobial Activity by Microdilution Assay

Minimum inhibitory concentration (MIC) and a minimal bactericidal concentration (MBC) of *V. floribundum* Kunth fruit extracts from different ripeness stages and heights were tested using the broth microdilution technique according to the Clinical and Laboratory Standards Institute’s [35] recommendations with some modifications. Single colonies, from a 24 h culture, were selected to prepare an inoculum corresponding to 1.5 × 10^8^ CFU/mL using a 0.5 McFarland standard turbidity.

The suspensions were adjusted for a final microorganism density of 5 × 10^5^ CFU/mL. Working solutions of raw fruit extracts were prepared by diluting the stock solutions with Mueller Hinton Broth (MHB) for a final concentration of 10 mg/mL. A 96-well U-shaped microplate was filled in the first wells with 100 µL of *V. floribundum* Kunth fruit extracts at different ripeness stages and allowed methanol evaporation for 30 min. Next, 100 µL of MHB was added to the first well and mixed. Next, 100 µL was taken from the first well to carry out 2-fold serial dilutions. After that, 100 µL of each bacterial suspension previously prepared was added to each well. The microplate was incubated at 37 °C for 24 h. The final concentrations of *V. floribundum* Kunth fruit extracts were 0.31–10 mg/mL. Methanol/water (80:20, *v*/*v*) was used as a negative control to discard any influence of methanolic solution in the inhibitory activity of the fruit extracts. This control was treated similarly to the samples. A well with MHB plus inoculum was used as a growth control, and a medium with no inoculum was applied to control sterility. Ciprofloxacin was used as a positive control with concentrations ranging from 0.09–3 µg/mL. The MIC was measured with the naked eye, comparing the results obtained with the negative control. The MIC is defined as the lowest concentration of a drug (extract) that inhibits the visible growth of an organism after overnight incubation [36].

MBC was determined following the MIC assay by plotting 3 µL of samples from clear wells onto NA plates without fruit extract. The agar plates were incubated at 37 °C for 24 h. MBC was estimated as the smallest sample concentration where no visible growth was present. MBC is defined as the minimum bacterial concentration required to completely kill the original inoculums. All MIC and MBC assays were performed in triplicate in at least two independent experiments.

### 2.9. Statistical Analysis

To perform the statistical analysis, the IBM SPSS Statistics software for Windows version 25.0 (SPSS Inc., Chicago, IL, USA) was used. The samples were analyzed in triplicate, and the means and standard deviations (SD) were used to report the results. One-way ANOVA and Bonferroni post hoc tests were used to statistically analyze the data between different groups. We used the grouped bar graph to express the results and the standard error. *p <* 0.05 was considered significant and *p* < 0.01 highly significant.

## 3. Results

### 3.1. Phenolic Profile of Andean blueberry (V. floribundum *Kunth*)

In this study, we analyzed the phytochemical composition of the wild Andean blueberry from two different altitudes by HPLC-DAD-ESI/MS^n^ at three different stages of maturation. In all cases, the extracts from Andean blueberries collected at the higher altitude during the three stages had higher concentrations of phenolic compounds than the extracts of the fruits from the lower altitude (Table 3). However, the phytochemical profile of all the extracts was the same regardless of the degree of maturity or altitude.

**Figure 2:**
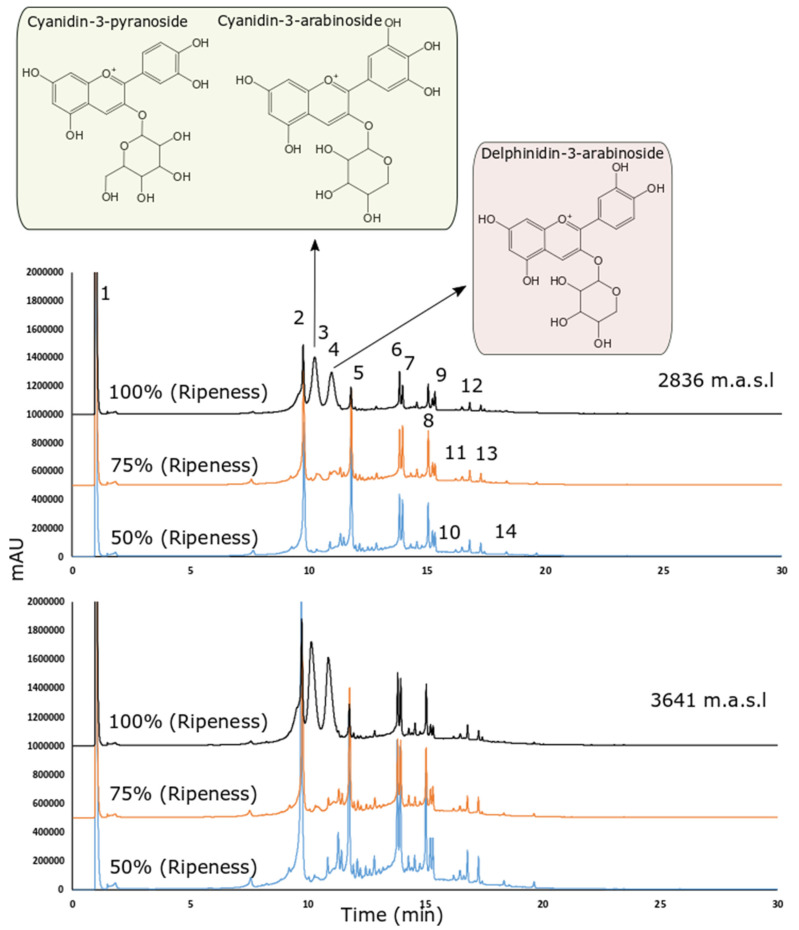
HPLC-DAD chromatogram recorded at 370 nm of the wild Andean blueberry (*V. floribundum* Kunth) fruit extract across different altitudes and ripening stages (blue: 50% ripeness, orange: 75% ripeness and black: 100% ripeness).

Figure 2 shows the chromatograms obtained under different altitudes and ripening stages. All tentatively identified peaks (shown in Figure 2) are presented in Table 3 based on the MS^2^ fragmentation patterns and their (pseudo) molecular ions, for which we relied on the available bibliography and our chromatographic peak library.

The masses 173 *m*/*z* and 191 *m*/*z* are consistent with quinic acid while the ruptures of 179 *m*/*z* in 135 and 161 are consistent with caffeic acid. Therefore, the parent masses 219 *m*/*z* and 225 *m*/*z* were tentatively identified as quinic and caffeic acid derivate (in this case a loss of 48 uma could indicate a carboxylic acid modification). A similar explanation regarding caffeic acid is also present in the 215 *m*/*z* detected in negative with low intensity at peak 2. The rupture in negative ionization mode was 353 *m*/*z* to 191 *m*/*z* (peak 2). The rupture pattern in positive ionization mode was found to be very similar to chlorogenic acid using the MzCloud database (best match with a similarity score of 89). However, the caffeoylquinic acid rupture in positive and negative is consistent with both spectra.

**Table 3:** Tentative Identification of Phenolic Compounds in *V. floribundum* Kunth by HPLC-DAD/ESI-MS^n^.

ID	Retention Time (min)	Precursor Mass (Negative)	MS/MS (Negative)	Precursor Mass (Positive)	MS/MS (Positive)	Identification	References
1	1.05	191	191-> 111(100), 173 (65), 127 (30)	193	193-> 147(100), 157(90), 175(20), 165 (15), 139 (5)	Quinic acid	[37]
		219	219-> 173(100), 191(70)	221	221-> 203(100), 157(90), 175(80	Quinic acid derivate	[37]
		225	225-> 179(100), 161(5) 179-> 135(100), 179(35), 161(30)			Caffeic acid derivate	[14,38]
2	9.78	353 (Low intensity)	353-> 191 (100)	355	355-> 163 (100), 145 (5)	Caffeoylquinic acid	[14]
		215 (Low Intensity)	215-> 179(100), 161(5) 179-> 135(100), 179(35), 161(30)			Caffeic acid derivate	[14,38]
3	10.26	-	-	449	449-> 287(100)	Cyanidin-3-pyranoside	[39]
		-	-	435	435-> 303(100)	Delphinidin-3-arabinoside	[39,40]
4	10.98	-	-	419	419-> 287(100)	Cyanidin-3-arabinoside	[39,40]
5	11.76	671	671-> 335 (100) 335-> 179(100), 135(20), 161(5)	-	-	5-*O*-Caffeoylshikimic acid	[3,14,37]
		335	335-> 179(100), 135(20)	337	337-> 163 (100), 145 (5)	5-*O*-Caffeoylshikimic acid	[37]
6	13.83	433	433-> 323(100), 161(50), 221 (16), 179(14)	435	435-> 307(100), 163(90)	6-*O*-Caffeoylarbutin	[37,41]
7	13.99	463	463-> 301(100)	465	465-> 303(100) 303-> 257(100), 285 (70), 229 (65), 165(55)	Quercetin glucoside	[14,40,42]
8	15.03	433	433-> 301(100)	435	435-> 303(100) 303-> 257(100), 285 (70), 229 (65), 165(55)	Quercetin pentoside	[3,14,38,42]
9	15.25	447	447-> 301(100)	449	449-> 303(100), 287(30) 303-> 257(100), 285 (70), 229 (65), 165(55)	Quercetin rhamnoside	[14,38,40]
10	16.20	577	577->433(100), 475(70), 515(65), 301(60)		579-> 303 (100), 561(35), 345 (15)	Quercetin 3-pentoside derivate	[14,43]
11	16.49	475	475->433(100), 415(55), 161 (5)		477-> 459(100), 385(60), 423(50), 441 (50), 367(30), 163(20)	Not identified	N/A
12	16.82	475	475->433(100), 415(55), 161 (5)			Not identified	N/A
13	17.23	591	591->447(100), 489(60), 529(20),301(5)		593-> 303(100), 413(50), 345(25), 575(20)	Quercetin hydroxymethilglutaryl- a-rhamnoside	[38]
14	18.32	301	301->179(100), 151(70), 273(15)	303	303-> 257(100), 285 (70), 229 (65), 165(55)	Quercetin	[37,42]

Peaks 3, and 4 correspond to ruptures of parent mass consistent with the anthocyanins. These ruptures were previously described in [39]. The parent mass 433 *m*/*z* in negative ion mode was reported in [41] as a caffeic acid derivate. However, the MzCloud reported a best match hit in positive ion mode (score of 88.1) with 6-*O*-Caffeoylarbutin (which is a caffeic acid derivate as reported in the publication). Peak 10 also showed 433 *m*/*z* and 301 m/z, consistent with quercetin pentoside (peak 8). However, the other ruptures are consistent with carboxylic acids, and a loss of 144 is possibly related to the loss of [Gal-2H_2_O] connected to hexose [43].

It is important to indicate that in Figure 2, the initial extract concentration as well as the injection volume was the same for all samples. Even when the primary goal was to perform a tentative identification and qualitative comparison, the peak areas also seem consistent with molecule concentrations.

### 3.2. Reducing capacity (RC), Total Flavonoids Content (TFC), Total Anthocyanin Content (ACY), Ascorbic Acid (AsA), and Total Free Amino acids (FAAs) of Andean blueberry (V. floribundum *Kunth*) with Respect to Altitude and Ripeness

The highest levels of RC were found at the higher altitude within each ripeness stage. Furthermore, the highest RC was found at 3641 m.a.s.l. in ripeness stage 3 while the lowest amount was reported for ripeness stage 1 at 2836 m.a.s.l. (Table 4).

**Figure 3:**
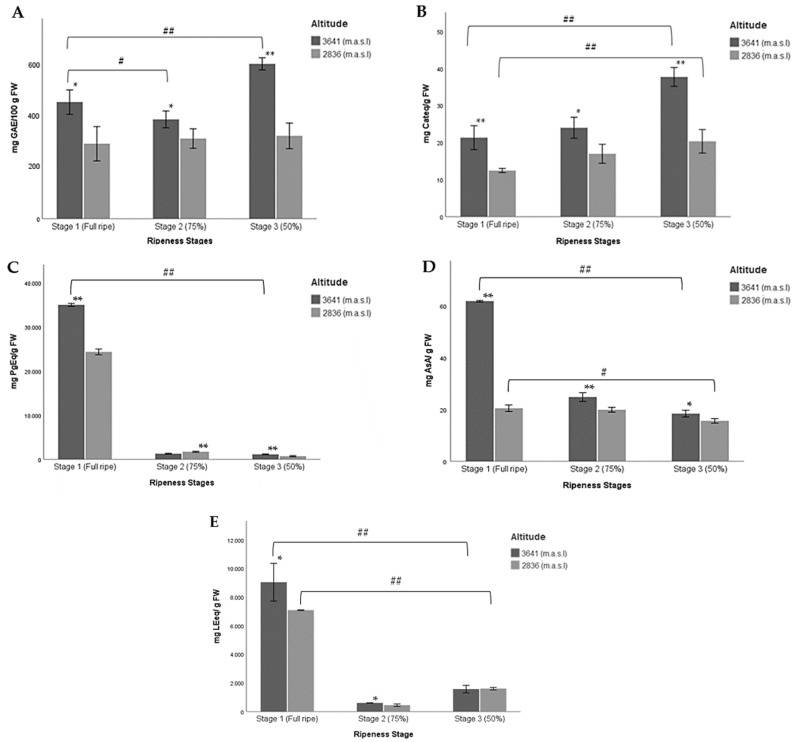
Multiple comparisons of three ripeness stages of Andean blueberries (*V. floribundum* Kunth) based on their pigmentation (Stage 1: fully ripe, stage 2: 75% completely pigmented, and stage 3: less than 50% pigmented) with respect to a high (3641 m.a.s.l.) and low (2836 m.a.sl) altitudes for the chemical composition of fruits. (**A**) Reducing capacity, (**B**) total flavonoid content, (**C**) total anthocyanin content, (**D**) ascorbic acid content, and (**E**) total amino acid content. The results are reported as mean ± SD of three experiments. * *p* < 0.05, ** *p* < 0.01, significant differences compared to low altitude; ^#^ *p <* 0.05, ^##^ *p* < 0.01, significant differences between the groups of ripeness stages at the lower and higher altitudes.

Figure 3 shows the values of the multiple comparisons made between the ripeness stages at both altitudes for the chemical composition of wild Andean blueberries. A significant difference between ripeness stages 1 and 3 at the higher altitude was found. It should be noted that at lower altitudes, no statistical differences were found for any of the ripeness stages (Figure 3A). In this sense, a significant decrease in the RC was observed when the berry begins to ripen and goes from a green/reddish pigmentation (less than 50%) to an almost completely pigmented berry (more than 75%) and finally ends up as a fully ripe berry (Table 4, Figure 3A).

In the case of TFC, the mean at 3641 m.a.s.l at ripeness stage 3 was higher than at 2836 m.a.s.l when the fruits were fully ripe, suggesting that TFC decreases with altitude as the fruit ripening process progresses (Table 4, Figure 3B). In the case of RC, we did not detect significant differences between ripeness stages 1 and 2. However, when comparing the three ripeness stages with each other at the lowest altitude, we found significant differences between stages 1 and 3 (Figure 3B).

Table 4 and Figure 3C show values that demonstrate that the highest content of anthocyanins was determined in fully ripe fruits at the higher altitude. The concentrations for ripeness stages 2 and 3 were very low, for both altitudes. In addition, we detected that the concentrations of ACY were higher at 3641 m.a.s.l than at 2836 m.a.s.l with respect to the three ripeness stages. No significant differences were found between maturation stages 2 and 3 at the higher altitude, yet we detected significant differences for the three stages at the lower altitude (Figure 3C). In general, the results suggest that ACY content in the wild Andean blueberry increases as the ripening process progresses.

The lowest concentrations of ascorbic acid were reported in ripeness stage 3 at the lower and higher altitudes (Table 4). Figure 3D presents similar levels of ascorbic acid in ripeness stages 2 and 3 regardless of altitude. These concentrations were three times lower than the concentration reported for stage 1 at the higher altitude. Significant differences were found in all cases. In this sense, a significant increase in the concentration of ascorbic acid was observed when the berries cultivated at the higher altitude began to ripen.

Finally, the results for free amino acid content were very similar to those for anthocyanins. We detected significant differences when we compared the ripeness stages with each other for the two altitudes. According to these data, a significant increase in FAA content was observed as the berry’s ripeness increased at the higher altitude (Table 4, Figure 3E).

### 3.3. Antioxidant Activity of Andean blueberry (V. floribundum *Kunth*) in Relation to Altitude and Ripeness Stage

**Figure 4:**
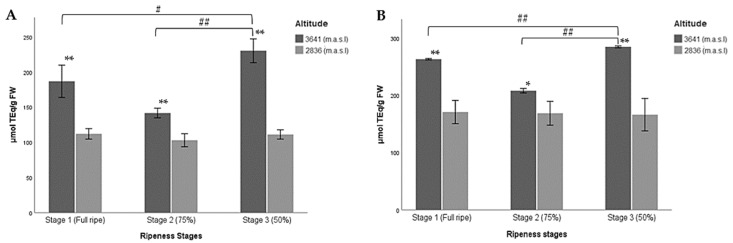
Multiple comparisons of the three ripeness stages of Andean blueberries (*V. floribundum* Kunth) based on their pigmentation (Stage 1: fully ripe, stage 2: 75% completely pigmented, and stage 3: less than 50% pigmented) with respect to the higher (3641 m.a.s.l.) and lower (2836 m.a.sl) altitudes for antioxidant capacity of fruits. (**A**) FRAP assay, (**B**) DPPH assay. The results are reported as mean ± SD of three experiments. * *p* < 0.05, ** *p* < 0.01, significant differences compared to the lower altitude; ^#^ *p* < 0.05, ^##^ *p* < 0.01, significant differences between the groups of ripeness stages at the lower and higher altitudes.

According to the results presented here, the hydroalcoholic extract of the wild Andean blueberries collected at ripeness stage 3 at the higher altitude showed the highest values of antioxidant activity according to the FRAP method (Table 5; Figure 4A). This coincides with the results reported here for RC and TFC (Table 4).

No significant differences were found between the ripeness stages of the lower altitude. However, significant differences were detected when the three stages of maturity were compared at the higher altitude. The lowest values were reported for ripeness stage 2 for both altitudes (Table 5; Figure 4A). These results suggest that ferric reducing/antioxidant power increased as altitude increased, and fruit ripeness decreased. At the lower altitude, total antioxidant activity remained constant throughout the three ripeness stages.

In the case of the DPPH assay, a higher antioxidant capacity was also found for the samples collected at the higher altitude at ripeness stage 3 and the lowest concentration in the lower altitude berries at the same ripeness stage (Table 5). Significant differences were found between the ripeness stages at the higher altitude when comparing all groups with each other, while for the lower altitude no significant differences were found between the ripeness stages.

Greater DPPH capacity was found in the berries at the higher altitude than in those at the lower altitude. The former had the highest values in all the stages studied (Figure 4B). In this sense, these results coincide with those reported for the FRAP assay, where the antioxidant capacity increased when the unripe berry developed at the higher altitude.

### 3.4. Antimicrobial Activity of Andean blueberry (V. floribundum *Kunth*) in Relation to Altitude and Ripeness Stage

The antimicrobial activity of *V. floribundum* Kunth was tested against a set of eight susceptible bacterial strains of clinical importance. A maximum initial concentration of the crude extracts was set at 10 mg/mL according to the results of previous tests (data not shown). The in vitro susceptibility tests showed that the *V. floribundum* Kunth fruit extracts at different ripeness stages and altitudes had different inhibition effects against gram-positive bacteria, including *E. faecium, E. faecalis,* and *S. aureus*, with MIC values that ranged between 1.3 and 10 mg/mL (Table 6).

A higher inhibition effect on gram-positive bacteria was observed on fruit extracts at ripeness stages 2 and 3 compared with extracts at ripeness stage 1. Extracts from stage 3 showed the best antimicrobial activities, with MIC values equal to or lower than 2.5 mg/mL. In addition, the extracts of fruits collected at the higher altitude showed better antimicrobial capacity against gram-positive bacteria than the samples collected at the lower altitude (Table 6). Thus, the best antimicrobial activity against the growth of *E. faecium, E. faecalis,* and *S. aureus* was found in samples at ripeness stage 3 and collected at the higher altitude, with a MIC value of 1.3 mg/mL.

A. *baumannii*, *P. aeruginosa, E. cloacae*, and *E. coli* were observed only at a MIC value of 10 mg/mL, mostly with samples from ripeness stage 3 and regardless of the sample’s altitude. No inhibitory activity was observed for *K. pneumoniae* (Table 6). Of note, extracts belonging to ripeness stage 1 had no inhibitory activity against the growth of gram-negative bacteria.

**Table 6:** Minimum inhibitory concentrations (MICs) and minimal bactericidal concentrations (MBCs), expressed in mg/mL of *V. floribundum* Kunth fruit extracts against bacterial strains of clinical importance. Ciprofloxacin (CP), expressed in µg/mL, was used as a positive control for all bacterial strains.

Susceptible Bacterial Strain	*V. floribundum* Kunth Extracts (mg/mL)												
	2836 (m.a.s.l)						3641 (m.a.s.l)						
	Stage 1 MIC MBC		Stage 2 MIC MBC		Stage 3 MIC MBC		Stage 1 MIC MBC		Stage 2 MIC MBC		Stage 3 MIC MBC		CP MIC (µg/mL)
*Enterococcus faecium* ATCC27270	10	10	5	5	2.5	2.5	5	10	2.5	2.5	1.3	2.5	1.5
*Enterococcus* *faecalis* ATCC29212	5	10	2.5	5	2.5	2.5	5	10	1.3	2.5	1.3	2.5	1.5
*Staphylococcus* *aureus* ATCC25923	5	5	2.5	5	2.5	5	2.5	5	1.3	2.5	1.3	2.5	0.4
*Klebsiella* *pneumoniae* ATCCBAA 700603	>10	-	>10	-	>10	-	>10	-	>10	-	>10	-	0.2
*Acinetobacter baumannii* ATCC19606	>10	-	10	-	10	-	>10	-	10	-	10	-	0.8
*Pseudomonas* *aeruginosa* ATCC27853	>10	-	10	-	10	-	>10	-	>10	-	10	-	0.4
*Enterobacter* *cloacae* ATCC23355	>10	-	10	-	10	-	>10	-	10	-	10	-	<0.09
*Escherichia coli* ATCC25922	>10	-	>10	-	10	-	>10	-	>10	-	>10	-	<0.09

The MBC/MIC ratio of *V. floribundum* Kunth fruit extracts against the gram-positive bacteria showed bactericidal antimicrobial activity. The MBC/MIC ratio indicates the type of antimicrobial activity; thus, for bactericidal drugs a value of less than or equal to four is expected, while for bacteriostatic drugs a value of more than four is expected [44]. The methanol/water solution used as a negative control showed no inhibitory effect on bacterial growth, discarding any influence of methanol in the inhibitory activity of the fruit extracts. MIC values of the positive control ciprofloxacin were as recommended, within plus or minus one or two-fold dilution of the expected MIC [36,45].

## 4. Discussion

### 4.1. Phenolic Profile of the Andean blueberry (V. floribundum *Kunth*)

Up to 14 of the detected peaks were tentatively identified, corresponding to quinic acid (1), phenolic acid (3), anthocyanins (4), flavonols (6), and 6-*O*-caffeoylarbutin derivatives. Flavonols and anthocyanins correspond to most of the flavonoids identified in *V. floribundum* Kunth, in terms of both the peak areas and number of identifications. Similar results were previously reported in the same species [46,47] and other berries belonging to the genus *Vaccinium,* such as *Vaccinium corymbosum* [48] and *V. myrtillus* [49].

Among the various aglycones belonging to the flavanols subclass, quercetin derivatives were the most abundant compounds identified in the extracts. These results are in agreement with previous studies into the Andean blueberry, where researchers reported that approximately 97% of the total peak area of flavonoids belonged to this compound [30,47].

We identified four anthocyanins: two derived from cyanidins (Cyanidin-3-pyranoside and Cyanidin-3-arabinoside) and one from delphinidins (Delphinidin-3-arabinoside). We can observe in Figure 2, that the abundance of anthocyanins is much higher in stage 1 (the highest stage of ripeness), which is consistent with our results regarding anthocyanin quantification (Table 4). Two studies into *V. floribundum* Kunth. determined that cyanidin derivatives were by far the most predominant anthocyanins (70.70–80%) in this species, followed by delphinidin derivatives (14.9–19%) [14,47,50]. The accumulation of cyanidins and delphinidin has been related to the deep purple-black color of berries, these contents being affected by the ripeness stage of the fruits and their growth conditions. These results agree with the distribution of anthocyanins described in this study (Figure 2 and Table 4). However, pelargonidin derivatives were not identified by those authors, as in our study. Another study coincides with our results and mentions that pelargonidin and malvidin glycoconjugates were identified in another species of the genus *Vaccinium* but not in *V. floribundum* Kunth. This could be a consequence of agronomic, genetic, and geographical factors. In addition, environmental factors such as sun irradiation, the difference between day and night temperatures, and direct sunlight intensity have also been shown to influence the metabolism of anthocyanins [3].

Regarding phenolic acids, it is worth noting that both caffeic acid derivatives and caffeoyl arbutin had high peak areas in this study. In fact, a recent study on *V. floribundum* Kunth reported that phenolic acids make up 15.7% of the total phenolic compounds, mainly represented by caffeoylquinic acids [14]. This agrees with the prevalence of phenolic acids in *Vaccinium* species reported in previous studies [47]. Studies into *V. corymbosum* and *V. myrtillus* pointed to caffeic acid as one of the main phenolic acids in berries. In addition, they emphasized its beneficial properties for health, not only its powerful anti-inflammatory, anticancer, and antibacterial power but also its antioxidant capacity [51,52]. Additionally, caffeoyl arbutin is known for its efficacy in the treatment of urinary tract infections and its skin-whitening properties [37].

### 4.2. Reducing Capacity (RC), Total Flavonoids Content (TFC), Total Anthocyanin Content (ACY), and Ascorbic Acid Content (AsA) of the Andean blueberry (V. floribundum *Kunth*)

Several studies have determined the physical-chemical composition and antioxidant capacity of the Andean blueberry (*V. floribundum* Kunth) [3,4,9,12,13]. However, there are no studies that evaluate the influence of altitude and state of fruit ripeness on the composition and concentration of phenolic compounds and biological capacities of the wild Andean blueberry, as well as the specific qualitative characteristics of the fruit.

The Folin-Ciocalteu (FC) assay results were interpreted as the RC of the samples according to the suggestion of several investigations [53]. The FC assay is based on electron transfer mechanisms that measure the ability of an antioxidant to reduce an oxidant, which changes color when reduced. The degree of color change correlates with the concentrations of antioxidants in the sample. This interpretation is also based on the results of other research projects, suggesting that the FC reagent reacts not only with the phenolic compounds but also with some amino acids, peptides, reduction sugars, ascorbic acid, Maillard reaction products, and other compounds in samples. Therefore, the interpretation of this test cannot be strictly linked to the content of polyphenols, but rather to the reduction capacity of the various components of the sample [53].

According to our results, the RC values of the fully ripe fruits from Carchi (at 2836 m.a.s.l) and Imbabura (at 3641 m.a.s.l) were 2894.94 mg GAE/100 g FW and 4503.78 mg GAE/100 g FW, respectively (Table 4). The results obtained coincide with results reported in previous studies of *V. floribundum* Kunth [54,55] and *Vaccinium glaucoalbum* [40]. Therefore, the results determine that the higher the altitude, the more RC there will be. Moreover, as previously indicated, even when the chromatograms were not obtained for quantitative purposes, we can notice an increment in the peak areas in the anthocyanin as well as other polyphenols identified (Figure 2), where values are greater at the higher altitude. This could support the idea that increases in polyphenols content could be partially responsible for the increment in the RC (Table 4). Similar results have been reported in other studies. One evaluated the content of polyphenols in *V. corymbosum* L. in two crops at two different altitudes, finding no clear effect of the influence of altitude. However, a similar pattern was evidenced in terms of the composition of polyphenolic compounds but not in concentration, since it was higher at higher altitudes [54]. Meanwhile, a study into strawberries reported that altitude significantly affected the concentration of single classes of phenolic compounds [24,40]. Particularly, the concentrations of caffeic acid derivatives increased with the rising altitude [45,46] as a response to higher sunlight spectra, visible light, and UV radiation [37].

Furthermore, a previous study suggested that caffeic acid derivatives are effective ROS (reactive oxygen species) scavengers and UV-absorbers as they contribute to cell wall thickening, which provides additional protection against the penetration of UV wavelengths [56]. One study in China determined that when the altitude increased from 2000 m to 3500 m, the contents of all the phenolic acids clearly increased, especially caffeic acid. They detected that this content was affected when plants were grown in a mountainous region at high altitudes, with values ca. 4-fold higher than those obtained at lower altitudes [57,58]. However, levels of hydroxycinnamic acids may vary. Previous research into *Vaccinium* berries reported a decrease in these compounds at later stages of berry development and a higher concentration in young fruits at high altitudes [53,59]. These results agree with the results here reported between ripeness stages 3 and 1 at the higher altitude (Figure 3A).

The TFC concentration range of the fruits at different altitudes and ripeness stages ranged from 12.44 to 37.71 mg Cateq/g FW (Table 4). Previous studies on *V. floribundum* Kunth [43,44], *V. corymbosum* L. [60], and *Vaccinium vitis idaea* L. [61] corroborate our results. According to the results obtained, TFC was also affected by the different altitudes of the production sites and the ripeness stage. We found that the high TFC in the wild Andean blueberry was positively associated with altitude. We conclude that higher altitudes, where there is a higher incidence of solar radiation, lead to elevated levels of TFCs, such as that of quercetin glycosides. According to previous research, the variation of the flavonoid concentration is closely related to altitude and the environmental conditions that it creates [62,63]. One study suggested that flavonoids play multiple roles in photoprotection. Therefore, they hypothesized that flavonoids accumulate in plants to inhibit ROS generation and then reduce ROS that have already been formed in response to stress produced by high levels of solar irradiation. It is worth noting that high light irradiation (high altitude) upregulates the biosynthesis of flavonoids such as quercetin and luteolin, but not that of flavonoids such as kaempferol and apigenin [56]. Another study on *V. myrtillus* determined that concentrations of quercetin and rutin rose significantly with increasing altitude (higher solar radiation) [62]. In addition, other studies suggested that fruits growing in a low-light environment show lower phenolic content, particularly a low concentration of flavonols [64,65], especially the proportion of kaempferol [53]. It is worth mentioning that altitude not only has a positive influence on the phenological moment of the fruit but also on its ripening dynamics (62). This explains the results obtained in our investigation. We detected highly significant differences between ripeness stages 3 and 1 at the higher altitude (Figure 3B). Previous research suggested that environmental parameters, such as temperature (decreases with altitude) and photosynthesis active radiation (increases with altitude), vary according to altitude, and these variables in turn play an important role in fully ripening the berries [53]. This coincides with our data (Table 2) concerning the environmental conditions of the localities where the wild Andean blueberries were collected. In addition, TFC is affected by physiological processes that take place as the berries ripen, where the accumulation of these increased progressively throughout ripening [66]. During the ripening of the berries, many complex biochemical changes occur, leading to cell enlargement, sweetening, softening, and pigmentation of the fruit. Flavonoids such as flavonols, carotenoids, and chlorophylls are the predominant compounds in berries during the early stage of ripening when pigmentation changes from green to pink. Therefore, the chlorophylls degrade and the concentration of flavonols and flavanols begins to decrease [53,66,67].

By contrast, according to previous reports, the accumulation of anthocyanins increases as the ripening process progresses, and they are the main flavonoids in ripe berries [66]. This approach agrees with the results here exposed. It has been reported that anthocyanins are present in the pulp of the berry but mainly in its skin. As the berries begin to ripen, their composition begins to vary. In this sense, the anthocyanin profile changes, and these compounds could range from 13 to 27 different types of anthocyanins present, depending on the genotype. This anthocyanin profile is significantly more diverse than other berries, such as blackberries, which contain only 5–8 anthocyanins [68,69]. Previous research has identified that the high accumulation of ACY in the ripeness stage is related to the increase in the transcription levels of UDP-glucose flavonoid 3-*O*-glucosyltransferase (UFGT), dihydroflavonol 4-reductase (DFR), chalcone synthase (CHS), and anthocyanin synthase (ANS) [53]. In this sense, and considering our results, it can be suggested that ACY content was influenced not only by the ripeness of the fruit but also by the altitude and environmental conditions of each production site (Table 4; Figure 3C), in that the higher the altitude, the higher the anthocyanin content. When similar studies were performed on other species such as *V. glaucoalbum* [40] and *Vaccinium uliginosum* [70], a high ACY content was found in fruits collected at higher altitudes, which is in line with the results presented here. Likewise, our results also partially agree with the results reported in the mature fruits of *V. corymbosum* L. grown in the northern lowlands (low altitude) and in the Alps (high altitude) of Italy. In addition, another research group evaluated the accumulation of anthocyanins in highbush blueberry (*V. Corymbosum* L.) for two years. They determined that the concentration of ACY increased 34-fold during maturation at higher altitudes, while at low altitudes the concentration increased only 25-fold [53]. This phenomenon may be a product of the plants’ protective mechanisms against extreme environmental conditions in high-altitude sites [62,71]. In fact, previous research suggested that UV radiation had less of an impact on anthocyanins than glycosylated flavonols. This diversity of responses is consistent with the fact that not all the gene sequences encoding the enzyme flavonol-synthase may be overexpressed by the UV doses received [72]. However, other studies determined that the accumulation of anthocyanins in the berries is influenced by temperature (a poorly recognized but essential environmental factor in the final chemical composition of the fruit [69,72]), dry matter, sugar content, organic acid content, firmness and oil content of the fruit [59]. Thus, some authors suggest that during ripening, daytime temperatures seem to be a determining environmental factor in the accumulation of anthocyanins in berries [57].

*Vaccinium* berries are also an important source of vitamin C. This antioxidant compound is a plant derivative, along with dehydroascorbic acid (oxidized form of AsA). Humans are not able to biosynthesize this compound; thus, AsA represents an essential vitamin in our diet [73]. One study in northern Italy evaluated the effect of ripeness and altitude on Vit C concentration in two cultivars of *V. corymbosum.* In both, the Vit C content was positively influenced by altitude throughout ripening. The ripe fruit of the first cultivar exhibited a 41% and the second cultivar a 35% higher vitamin C content with respect to less ripe fruits cultivated at a high altitude. However, no significant changes were found at a low altitude, where Vit C content remained relatively stable along the ripening process [57]. Although the genotype of the plants is the main factor that determines the concentration of Vit C in the berries, levels of ascorbic acid are also influenced by different environmental factors, such as solar light [73]. In response to intense light, the massive vacuolar accumulation of AsA increases sharply [56]. In fact, previous studies determined that AsA content increased throughout the ripening process [57]. Nonetheless, the concentration of AsA decreased in overripe berries [74], as with our results (Table 4; Figure 3D). In contrast, one study determined that the levels of AsA stayed relatively stable over *V. myrtillus* L.’s ripening process. Little is known about the oxidation, recycling, and molecular mechanisms that regulate AsA biosynthesis during the development and ripening of *Vaccinium* berries [73].

Finally, the metabolism of free amino acids changes throughout the life of the plants, either due to environmental conditions, phenology, and physiology or the interaction between these and the plants’ metabolic pathways [75,76]. The variation that was found in FAAs between the different ripeness stages and altitudes could be due to the physiological changes that occur in the fruits during ripening. Indeed, the highest content was founded in fully ripe berries at the higher altitude (Table 4, Figure 3E). This could be because these compounds in the plant tissues increase in response to abiotic stress, which the plants experienced with greater force throughout their development at higher altitudes [77,78]. In the metabolism of plants, these compounds have an adaptive capacity, changing according to present conditions. This was demonstrated in a study carried out in *Arabidopsis* plants, where the composition of FAAs changed according to the severity of water stress that their seeds suffered [79]. On the other hand, proline levels increased when plants were exposed to stress factors such as drought, UV radiation, cold, high salinity, and pathogen infections [80]. FAAs play an essential role in the growth and development of organisms [81]. Some of these act as precursors of primary and secondary metabolites, which apart from being involved in primary metabolic functions also protect the plant [79]. Few studies report data on FAAs; most research focuses on ACY, leaving amino acids aside, even though they could contribute to the nutritional content of berry extracts [82].

### 4.3. Antioxidant Activity of Andean blueberry (V. floribundum *Kunth*) in Relation to Altitude and Ripeness Stage

Previous studies have shown that the concentration of polyphenols can increase in response to oxidative stress caused by various factors, such as pests, diseases, ultraviolet light, low temperatures, soil nutrients, and drought [83,84]. Our results for antioxidant capacity are consistent with those and other studies conducted on the same berries species collected in the Ecuadorian Andean region [4,54]. These show that the highest antioxidant capacity is found in fruits collected at high altitudes (Table 5), which could be related to the climatic conditions present there (Table 2), such as greater solar radiation [65,85]. In fact, one study suggested that this could be interpreted as a protective regulatory response that increases both UV sensing and antioxidant capacity. These processes can be interpreted as “good stress” instead of anguish (“destructive stress”) [72]. In studies carried out on raspberries and strawberries, it was found that fruits from organic crops had higher values of antioxidant activity, since they are exposed to greater environmental stress than traditional crops [86,87]. One study carried out on *V. myrtillus* reported higher values of antioxidant activity and phenolic compounds in northern European, growing in environments with lower temperatures, than blueberries from countries located further south with warmer temperatures [88]. The higher the latitudes, altitudes, and sunny weather, the higher the total polyphenol content will be in different *Vaccinium* species [89].

Antioxidant capacity also varies according to the ripeness stage of the fruits, which is related to the content of bioactive compounds that the berries present in their different stages of development, since these compounds provide an antioxidant effect to the berries [90]. In our study, a higher antioxidant capacity was reported at the higher altitude in less mature fruits (i.e., stage 3), followed by ripeness stage 1, and finally stage 2 (Table 5; Figure 4). The same pattern was observed in the RC. We believe that the antioxidant capacity of the wild Andean blueberry grown at a higher altitude is indeed related to the RC. A study conducted on five varieties of Korean blueberries found that antioxidant activity was higher in unripe fruit, and this was highly correlated with flavonoids and reducing capacity [91]. In addition, one investigation group reported a high positive correlation between RC and antioxidant activity in *V. myrtillus* samples from different geographical locations [88]. This was corroborated by a study in four Peruvian berries, including Andean blueberries (*V. floribundum* Kunth). The investigators emphasized that antioxidant capacity depends on structural factors of phenolic compounds, such as the position and number of methoxyl or hydroxyl groups in the phenolic ring [92]. The same results were reported in other studies on chokeberries [91,93] and strawberries [94]. However, negative correlations between antioxidant capacity and RC have been observed in other fruits such as guava [91]. For this reason, more research is needed to identify the compounds contributing to the antioxidant capacity of Andean blueberries. That said, the antioxidant capacity could vary not only due to maturity and altitude but also to the species and variety of the fruit [95].

### 4.4. Antimicrobial Activity of Andean blueberry (V. floribundum *Kunth*) in Relation to Altitude and Ripeness Stage

Our results showed that *V. floribundum* Kunth extracts have a better inhibition effect on the growth of gram-positive bacteria than on gram-negative bacteria (Table 6), and that the ripeness stages and harvest altitude also influence *V. floribundum* Kunth’s antimicrobial activity. This is the first study that correlates the antimicrobial activity of *V. floribundum* Kunth extracts with two factors (ripeness and harvest altitude).

Importantly, the best inhibition activity against the growth of *E. faecium*, *E. faecalis,* and *S. aureus*—with MIC values of 1.3 mg/mL (Table 6)—came from extracts collected from unripe fruits (stage 3) from high altitudes. Our data showed that these unripe fruits contained high concentrations of phenolic compounds, such as quercetin, quinic acid, caffeic acid, 5-*O*-Caffeoylshikimic acid, and 6-*O*-Caffeoylarbutin (Table 3). Phenolic compounds are well known for their antimicrobial activity [96], and the phenolic derivates found in this study have also been reported for their inhibitory properties over bacterial growth [97]. The inhibition mechanism is thought to be related to the number of hydroxyl groups present in phenolic compounds, which might interrupt the enzymatic activity of microorganisms [52,98,99]. In addition, it is presumed that the antimicrobial activity of these compounds is attributed to their reaction with intracellular molecules of the microorganisms and subsequent interference in cellular metabolism [100].

On the other hand, the antimicrobial activity of *V. floribundum* Kunth fruit extracts against gram-negative bacteria was only seen at a MIC value of 10 mg/mL, mostly with samples collected from unripe fruits and regardless of the harvest altitude. The main difference in chemical composition between ripeness stages found in this study is the amount of reducing capacity present; thus, at stage 3 there was a higher amount of RC and TFC compared with other levels of ripeness (stages 1 and 2) (Table 4). In addition, at the higher altitude, higher concentrations of these compounds were present. Therefore, these data confirm that the antimicrobial activity observed in both gram-positive and gram-negative bacteria is due to the high concentrations of phenolic compounds. The low inhibitory activity observed in the gram-negative bacteria may be due to the presence of an outer membrane of lipopolysaccharide that surrounds the cell wall of said bacteria. The absence of this membrane in gram-positive bacteria may contribute to a greater permeability of the bioactive phytochemicals of the wild Andean blueberry in the cells, leading to greater bacterial inhibition [13,97].

A previous study looking at the antimicrobial activity of *V. floribundum* Kunth extracts suggested that the high polyphenol levels in the extracts, especially phenolic acids, organic acids, and flavonol glycosides, inhibit both biofilm production and the growth of bacterial species [97]. However, they evaluated the antimicrobial activity using only the well-diffusion method and did not present MIC value, making any comparison with our study difficult. It has been strongly suggested that only MIC and MBC values should be published to facilitate analyses of the results of different studies [101]. Other studies with *Vaccinium macrocarpon* [100,102] and *V. meridionale* [13] evaluated the role of certain more complex phenolic polymers, such as proanthocyanidins. These compounds are flavonoids present in *Vaccinium* berries and are composed of (-)-epicatechin and/or (+)-catechin units linked by type A and type B interflavanic bonds (13). These studies linked the antibacterial activity against gram-negative and gram-positive bacteria to the action of type A proanthocyanidins. They determined that these compounds, when in contact with the bacterial membrane, increase their permeability, giving rise to subsequent perforation, disintegration, and cell death [88]. In this study, we were able to identify only four types of anthocyanins: delphinidin-3-pyranoside, cyanidin-3-pyranoside, delphinidin-3-arabinoside, and cyanidin-3-arabinoside (Table 3).

Overall, our data suggest that *V. floribundum* Kunth has great potential to become a source of useful antimicrobial agents in the pharmaceutical and food industries. However, further analyses are needed to clarify whether the antimicrobial activity of the extracts is attributed solely to a single bioactive compound or to the complementary, synergistic, and/or additive effects of multiple phytochemicals. This phenomenon will depend on many factors, including geographical and environmental conditions [102].

## 5. Conclusions

The Andean blueberry contains a unique combination of bioactive phytochemicals that play an important role in its characterization as a functional food. We determined that altitudinal gradients and the ripeness stages of *V. floribundum* Kunth influenced the concentrations of bioactive compounds, as well as the antioxidant capacity and antimicrobial activity. Particularly, the wild Andean blueberries collected in the upper zone presented higher concentrations in all analyses than those from the lower zone, regardless of the ripeness stage of the fruit. It is worth mentioning that, when the berries began to ripen, the concentration of RC, TFC, antioxidant capacity, and antimicrobial activity began to decrease. By contrast, the total content of ascorbic acid, FAAs, and ACY increased as the ripening process progressed. Climatic trends and geographical conditions are associated with altitude and play an important role not only in the ripening of the berries but also in the final phenolic profile of the fruits and their antimicrobial activity. Overall, our data provide evidence regarding the functional value of the wild Andean blueberry. Notwithstanding the importance of the results presented here, as a future perspective, new studies covering other geographical areas across the same altitude should be considered, as well as those that evaluate other biological activities related to polyphenols (e.g., anti-inflammatory and antitumor activities). This will undoubtedly provide more evidence allowing other conclusions on the effect of the altitude gradient and ripeness stages of the fruit on its functional potential.

## Figures and Tables

**Table 1:** Geographical description of the localities where the wild Andean blueberries (*V. floribundum* Kunth) were collected.

Province	Carchi	Imbabura
Canton	Montúfar	Ibarra
Parish	La Paz	Zuleta
Latitude	00°36.478′ N	00°13.523′ N
Length	77°49.716′ O	78°03.074′ O
Altitude (m.a.s.l)	2836	3641

**Table 2:** Environmental conditions of the localities where the fruits of the wild Andean blueberry (*V. floribundum* Kunth) were collected.

	Temperature (°C)	RH (%)	Dew Point (°C)	Atmospheric Pressure (hpa)	Winds (km/h)	Rain Events (mm)
Carchi						
Mean	10.21	86.69	7.93	708.08	1.62	7.95
Min	2.20	43.00	−7.50	659.70	0.00	0.00
Max	20.10	99.00	12.30	734.80	16.60	55.10
Zuleta						
Mean	8.62	59.14	7.05	688.48	3.92	4.63
Min	1.80	38.00	−7.50	659.70	0.00	0.00
Max	18.20	99.00	11.50	733.70	23.00	55.10

**Table 4:** Chemical composition of Andean blueberries (*V. floribundum* Kunth) as affected by site altitude and ripeness stage.

Parameter	Altitude	Stage of Ripeness			*p*-Value (Stages)
		Stage 1 *	Stage 2 *	Stage 3 *	
RC (mg GAE/100 g FW) ^1^	3641 (m.a.s.l)	4503.78 ± 409.60	3836.64 ± 281.6	5982.56 ± 202.80	<0.001
	2836 (m.a.s.l)	2894.94 ± 574.91	3093.64 ± 324.90	3199.61 ± 428.52	0.72
*p*-value (Altitude)		0.017	0.04	0.001	
TFC (mg Cateq/g FW) ^1^	3641 (m.a.s.l)	21.31 ± 2.79	23.98 ± 2.45	37.71 ± 2.20	<0.001
	2836 (m.a.s.l)	12.44 ± 0.48	16.94 ± 2.22	20.31 ± 2.75	0.01
*p*-value value (Altitude)		0.006	0.021	0.001	
ACY (mg PgEq/g FW) ^1^	3641 (m.a.s.l)	35,006.97 ± 270.90	1267.86 ± 75.90	1121.59 ± 74.50	<0.001
	2836 (m.a.s.l)	24,360.20 ± 558.72	1711.65 ± 114.03	705.67 ± 114.03	<0.001
*p*-value value (Altitude)		<0.001	0.005	0.006	
AsA (mg AsA/g FW) ^1^	3641 (m.a.s.l)	61.79 ± 0.24	24.81 ± 1.47	18.41 ± 1.12	<0.001
	2836 (m.a.s.l)	20.46 ± 1.08	19.89 ± 0.80	15.59 ± 0.75	0.001
*p*-value value (Altitude)		<0.001	0.01	0.02	
FAAs (mg LEeq/g FW) ^1^	3641 (m.a.s.l)	9040.02 ± 249.01	601.18 ± 11.82	1556.08 ± 225.72	<0.001
	2836 (m.a.s.l)	7087.45 ± 270.90	444.31 ± 70.71	1587.69 ± 74.81	<0.001
*p*-value value (Altitude)		0.04	0.02	0.83	

^1^ The results are expressed as mean ± SD. * (Stage 1: fully ripe, stage 2: 75% to completely pigmented, and stage 3: less than 50% pigmented). ACY: Total anthocyanins content, FAAs: Total free amino acid content, TFC: Total flavonoid content, RC: Reducing capacity, AsA: Ascorbic acid content.

**Table 5:** Antioxidant capacity of Andean blueberries (*V. floribundum* Kunth) as affected by site altitude and ripeness.

Parameter	Altitude	Ripeness Stages			*p*-Value (Stages)
		Stage 1 *	Stage 2 *	Stage 3 *	
FRAP (μmol TEq/g FW) ^1^	3641 (m.a.s.l)	186.68 ± 19.92	141.47 ± 5.90	230.15 ± 14.71	0.001
	2836 (m.a.s.l)	111.86 ± 6.48	102.76 ± 8.07	110.96± 5.67	0.27
*p*-value (Altitude)		0.003	0.003	0.001	
DPPH (μmol TEq/g FW) ^1^	3641 (m.a.s.l)	263.28 ± 1.14	208.01 ± 3.41	284.94 ± 1.43	<0.001
	2836 (m.a.s.l)	170.67 ± 17.70	168.56 ± 18.12	166.07 ± 24.61	0.96
*p*-value (Altitude)		0.001	0.02	0.001	

^1^ The results are expressed as mean ± SD. * (Stage 1: fully ripe, stage 2: 75% to completely pigmented, and stage 3: less than 50% pigmented). FRAP: Ferric reducing/antioxidant power test, DPPH: 2.2-diphenyl-1(2,4,6-trinitrophenyl) hydrazyl method.

## Data Availability

All data presented in this study are available on request by contacting the corresponding authors J.M.A.-S. and E.T.

## Supplementary Materials

The following supporting information can be downloaded at: https://www.mdpi.com/article/10.3390/molecules27217525/s1. Weather stations parameters in both locations.

## Sample Availability

Samples of fruits are available from the authors.
