# Supplementary material for: Influence of Altitudes and Development Stages on the Chemical Composition, Antioxidant, and Antimicrobial Capacity of the Wild Andean Blueberry (Vaccinium floribundum Kunth)
Source: Molecules. 2022 Nov 3;27(21):7525. doi: 10.3390/molecules27217525 (PMC9657399; doi:10.3390/molecules27217525)

Location: Carchi.

Altitude: 2836 m.a.s.l

Data recollected every 2h during 10 days after recollections

Temperature (oC)

1st. Collection (July) 2nd. Collection (September) 3rd. Collection (November)

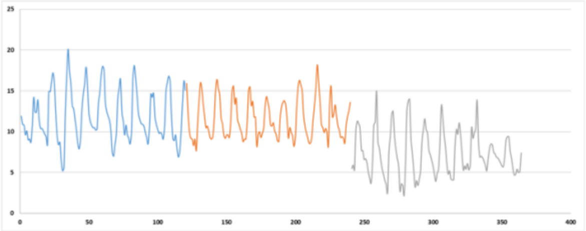

RH (%)

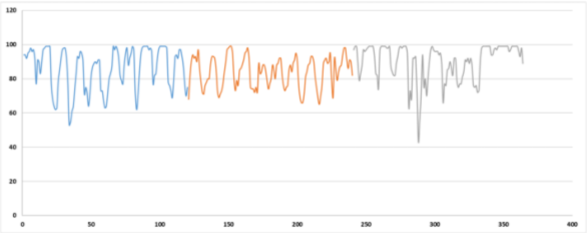

Dew Point(oC)

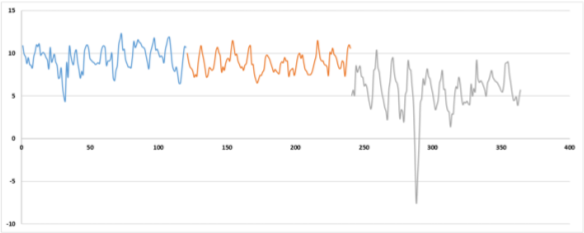

Atmospheric pressure (hpa)

1st. Collection (July) 2nd. Collection (September) 3rd. Collection (November)

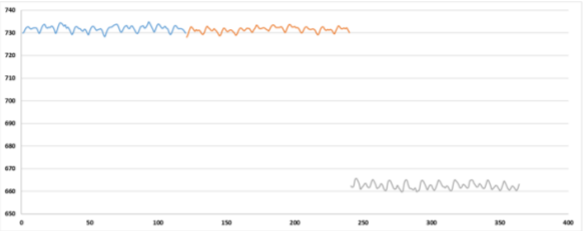

Winds (Km/h)

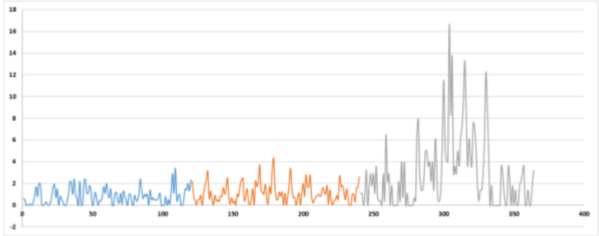

Rain Events (mm)

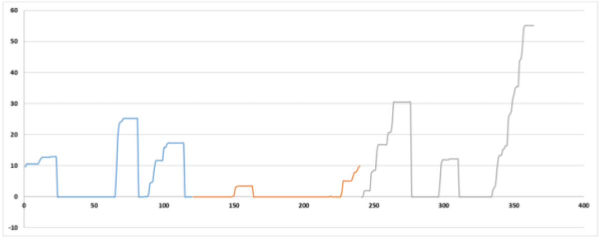

Location: Zuleta.

Altitude: 3641 m.a.s.l

Data recollected every 2h during 10 days after recollections

Temperature (oC)

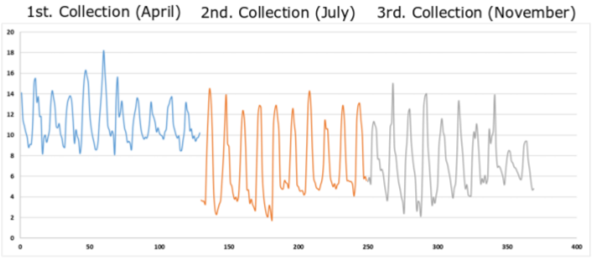

Atmospheric pressure (hpa)

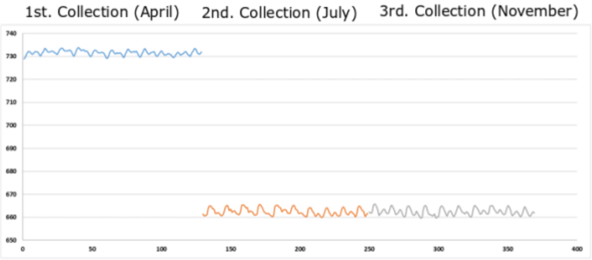

RH (%)

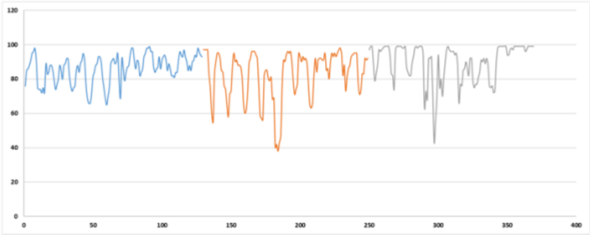

Winds (Km/h)

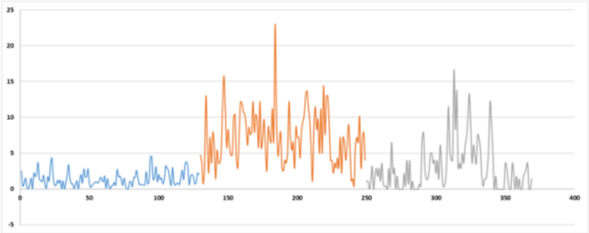

Dew Point(oC)

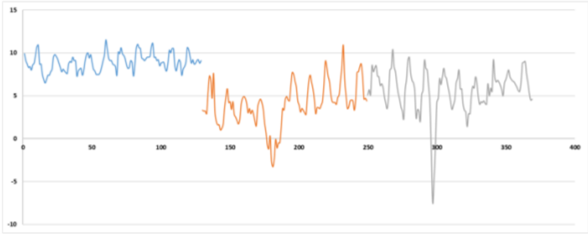

Rain Events (mm)

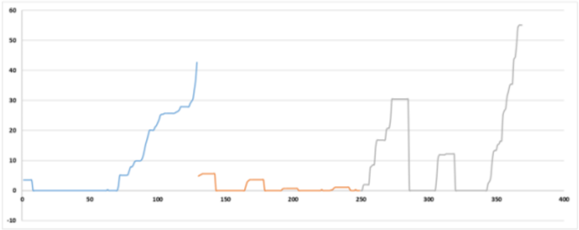

Supplement: Supplementary file 1 [file molecules-27-07525-s001.zip › molecules-1958491-supplementary.pdf]
